# Short and Tall Men

**Published:** 1891-10

**Authors:** 


### SHORT AND TALL MEN.

That a tall man is not necessarily talented is a fact of every day experience (says the London *Evening Standard*), and it has never been shown that there is essential connection between stature and statesmanship. If in Prince Bismarck we have an instance of a man of exceptional height, who is at the same time the foremost diplomatist of of his times, we find, on the other hand, that his most formidable

Parliamentary antagonist was "his little Excellency," the late Dr. Windthorst, a man of notoriously short stature, who raised himself from the humble position of a peasant's son to the rank of a Cabinet Minister and the greatest independent political leader in the new German Empire. Agamemnon was a warrior who stood head and shoulders above the Greeks before Troy; but Julius Cæsar and the First Napoleon are examples of the greatest generals who were distinguished by every thing but a commanding figure. Lord John Russel, of whom it was said that he was ready at any moment to form a Cabinet, or "take command of the Channel Fleet," was a dwarf beside Lord Palmerston, but his rival and peer in political eminence. While the leaders of men in the old world are thus pretty evenly balanced between the tall and the short, it has been reserved for the youngest nation of the world to furnish a remarkable instance of an assembly of the most eminent statesmen who were, at the same time, men of exceptional stature. The 44 gentlemen who, at their recent gathering in Sydney, succeeded in drafting the outlines of the Constitution of the great united Australian Commonwealth of the future, included all the Premiers, leading Ministers, and leaders of the Opposition of all the seven Australian colonies, and they were, almost without exception, tall men. Their average height was close upon six feet.

